# Lessons Learned Identifying and Controlling Fraudulent Participation in Online Randomized Trials

**DOI:** 10.2196/77512

**Published:** 2025-10-29

**Authors:** Robert Siebers, Kara M Magane, Hattie Slayton, Skylar Karzhevsky, Tibor P Palfai, Ana M Abrantes, Lisa M Quintiliani, Michael D Stein

**Affiliations:** 1 School of Public Health Boston University Boston, MA United States; 2 Department of Psychological and Brain Sciences Boston University Boston, MA United States; 3 Department of Psychiatry and Human Behavior Alpert Medical School Brown University Providence, RI United States; 4 Department of Medicine Tufts University Tufts Medical Center Boston, MA United States

**Keywords:** clinical trials as topic, fraud detection in clinical research, fraud, fraudulent research participation, HIV, virtual clinical trials

## Abstract

Virtually conducted clinical trials have become an important tool for improving access to research. Online research gives rise to new avenues for potentially fraudulent actors to participate in studies to achieve monetary gain. We describe our experience of uncovering and removing fraudulent participants from a virtual research study and our methods to prevent fraudulent participants in the future. Fraudulent participation in the 2 linked online clinical trials was first uncovered in 2023, prompting our investigation and identification of additional fraudulent participants (falsified identity or information to meet eligibility criteria) who successfully enrolled in these trials. Our study team categorized indicators of suspicious activity at prescreening, screening, and baseline stages of study participation and implemented a manual checklist method to prevent fraudulent participation. We evaluate the effectiveness of our fraud prevention methods 6 months after the initial breach of the trials. Before initial detection, 10 fraudulent participants successfully enrolled in our trials. Following the implementation of new fraud prevention measures, 37 individuals were identified as fraudulent at the screening stage, and no new fraudulent participants were enrolled. We provide a comprehensive list of suspicious behaviors that may suggest the virtual research intrusion of persons using fake identities. For online clinical studies, manual methods of fraud prevention, used in conjunction with automated prevention methods, can equip researchers to detect evolving patterns of attempted fraudulent enrollment.

## Introduction

Since the arrival of the COVID-19 pandemic, virtual approaches to clinical research have become more common. Virtual studies that involve digital advertising, internet-based screening, recruitment, and enrollment, as well as the use of videoconferencing for assessment and behavioral interventions, can improve access to research for marginalized and underrepresented communities. Online trials provide a way to enrich the geographical diversification of study participants and lower access barriers, such as travel expenses and time commitment [1].

Remote research also opens new avenues for fraudulent participation that in-person clinical trials rarely face. Fraudulent participation occurs when an individual who would otherwise be ineligible for a study misrepresents or falsifies their identity, health history, or other information to meet eligibility criteria, or participates in a study more than once [2]. Financial incentives can be a driving factor in the misrepresentation of personal history to gain enrollment in online research [3]. Previous research has suggested that deceptive or false responses on eligibility screenings to bypass otherwise restricting criteria are not rare in all paid research [4], and identity verification can be particularly difficult over the internet.

With the rise in remote research, fraudulent participation has become an increasing problem [5]. Investigators have used various methods to prevent fraud during the enrollment process. For instance, researchers have used IP address identification systems to determine online screening forms submitted from the same device so as to reduce the likelihood of a participant enrolling more than once [6-8]. Bot detection methods such as CAPTCHA and reCAPTCHA (Google Inc) have been effective in policing online survey research [1,7-11]. Suspicious activity, such as short response times on screening forms or surveys [6-8,11,12], a sudden influx of new screening form submissions [7,10,12,13], a screening-form response arriving in the middle of the night [10,11], or higher than expected enrollment in online surveys [10,12,14], has also been used to flag potentially fraudulent activity. Methods to cross-reference a potential participant’s identity with publicly available information have also been used [15].

Though these preventive approaches have been useful, some require financial and technological resources that are not always available. Moreover, with increasing sophistication and organization of those seeking to defraud studies, additional strategies may be needed to detect enrollment fraud in virtual studies.

This paper describes our experiences identifying fraudulent participants who enrolled or attempted to enroll in 2 online trials and the approaches we developed and used to address and prevent fraudulent activity.

## Overview of the 2 Remote Clinical Trials

The Boston ARCH Comorbidity Center, known as ARCHER (Addressing Related Comorbidities for HIV by Employing Remote Technologies), is a research center funded by the National Institute on Alcohol Abuse and Alcoholism. ARCHER conducts 2 eHealth clinical trials, the Integrated Telehealth Intervention to Reduce Chronic Pain and Unhealthy Drinking Among People Living With HIV [16] (hereafter the “Pain trial”; ClinicalTrials.gov NCT05503173) and the Increasing Physical Activity Among People Living With HIV Engaged in Unhealthy Drinking [17] (hereafter the “PA trial”; ClinicalTrials.gov NCT05505942). Through randomized controlled remote interventions, the Pain and PA trials aim to address 2 known comorbidities for HIV—chronic pain and physical inactivity [18-22]. Over the course of an approximately 6-month study period, participants complete standardized interview assessments administered by study staff and ecological momentary assessments (EMA) via a mobile phone app (Metricwire), and PA trial participants are also mailed a Fitbit to measure physical activity. Participants can receive up to US $480 as compensation for their participation; compensation is distributed in gift certificates following the completion of each planned study activity. Participants are recruited virtually from metropolitan areas in the United States with high HIV prevalence in collaboration with BuildClinical, a company that specializes in targeted online advertising for research recruitment.

Potential participants who find a BuildClinical digital advertisement and are interested in our studies are directed to a study website hosted by BuildClinical, where they complete an online prescreening form with questions to determine initial eligibility for the 2 trials. The form asks for name, phone number, email address, age, sex, ZIP code, race, HIV status, questions about recent alcohol consumption behaviors, physical activity behaviors, pain, interest in pain management, interest in increasing physical activity, and availability for a 15-minute phone screening.

Participants are initially eligible if they are (1) aged at least 18 years, (2) HIV positive, (3) engage in unhealthy drinking, and (4) rate their pain level as 4 or more (out of 10) for at least 3 months or report engaging in less than 150 minutes of moderate to vigorous physical activity per week for at least the past 3 months. Based on responses to eligibility criterion 4, potential participants may be initially eligible for only one trial or both the Pain and PA trials.

Potential participants identified as initially eligible are contacted by phone for further screening to determine their eligibility for trial enrollment. Potential participants who are initially eligible for both trials based on their prescreen responses are phone-screened for the Pain trial if they express a higher interest in pain management as compared to interest in increasing physical activity, and vice versa for the PA trial. Additional inclusion criteria assessed at phone screening include residing in the United States, having a US mailing address, and being willing to provide an alternative contact person to assist with study follow-up. Exclusion criteria assessed during phone screening include having a history of bipolar disorder, schizophrenia, schizoaffective disorder, or mania; a history of withdrawal-related seizures or delirium tremens; medical contraindications for physical activity (PA trial); or current nonpharmacological treatment for chronic pain (Pain trial).

If potential participants are deemed fully eligible during the phone screen and are interested in participating, a videoconference is scheduled during which participants provide informed consent, verify their HIV diagnosis by showing either a bottle of their HIV medication with their name on it or a copy of their medical record, and complete the baseline assessment. Both trials have been approved by the Boston University Medical Campus Institutional Review Board (IRB). The PA trial received initial IRB approval on December 16, 2022, and the Pain trial received initial IRB approval on December 21, 2022.

Between February 2023 and November 20, 2024, a total of 2626 potential participants completed a prescreening form, of which 1945 were initially eligible for either or both the Pain and PA trials. Of those initially eligible, 623 completed phone screening for the Pain trial, with 247 determined eligible, and 599 completed phone screening for the PA trial, with 258 determined eligible. Enrollment in these 2 trials is ongoing.

## Uncovering Suspicious Activity

In October 2023, a research assistant (RA) suspected they were conducting a videoconference informed consent for the Pain trial with the same person with whom they had completed a consent and baseline assessment 5 days prior. The person appeared to be wearing a wig during this second encounter. When asked to provide a prescription or medical record verification of an HIV diagnosis, this person provided a photograph on their phone of a paper copy of a medical record. The RA noted that the Android phone with the image of the paper medical record looked to be the same as the one they had seen 5 days previously, and the phone’s text notably displayed the same unusual curly-style font. In consultation with the project manager, baseline data were not collected for this participant, and the baseline assessment was terminated.

After this event, the study team convened to discuss this occurrence. Through this conversation, the study team uncovered peculiar, shared experiences among RAs who had conducted baseline assessments in recent weeks. The team noted a recent increase in the frequency of individuals completing baseline assessments with their camera off and confirming their HIV diagnosis with a paper copy of their medical record, both of which had been rare during the previous 8 months of study screening. Due to the diverse national sample being recruited, it was not uncommon for RAs to interact with participants who had accents. However, RAs noted that recently they had seen an increase in individuals with accents never heard previously. Given these initial unusual observations, the team decided to closely examine data at each step of the 2 trials’ recruitment, screening, and enrollment process for potential participant fraud.

RAs reviewed data from online prescreening forms that had been submitted in the preceding weeks of October 2023 and noticed various patterns that were out of the ordinary. The team noted a recent increase in respondents reporting first names (eg, John Mark) as their full name, email addresses that were Gmail accounts that followed a predictable pattern (first name, last name, string of digits), completed prescreening forms from individuals aged 20-35 years, forms that had ZIP codes and area codes that did not correspond to the same location, a higher number of forms that indicated male sex, forms indicating high levels of physical activity (ie, hundreds of minutes per day), forms that indicated strong interest (5 on a 5-point scale) for both studies, and forms that indicated the individual was available for a phone screening call at any time of any day. None of these prescreening form responses was suspicious in isolation; rather, the increasing numbers and combinations of these unusual characteristics raised suspicion among the study team.

When called to complete a phone screen, individuals who were suspected of being fraudulent based on the prescreening patterns described above frequently used a Google Voice phone number. This was evident in an automated voice assistant and answering machine that is shared among Google Voice numbers. Google Voice allows individuals to make internet-based phone calls from around the world and permits the creation of numerous phone numbers on a single device.

Many suspected fraudulent persons would urgently call or email the study numerous times if they missed the study staff’s attempted phone screening call. When reached, suspect individuals often tried to rush or skip the reading of the brief screening agreement and never asked follow-up questions when prompted. All suspicious individuals reported living in New York, Texas, or California and would occasionally provide a ZIP code or age during the phone screen that did not match their responses on their online prescreen form. These individuals would also provide quick responses to questions that often required more thoughtful responses from most other screened persons.

The study team then reviewed the Metricwire (the program used for EMA data collection) activity log of every participant enrolled in both trials who had completed or were actively engaged in EMA. The team discovered that 10 participants had connected to a virtual private network that was in a UTC +1 time zone outside of the United States in order to complete their EMA. Because Metricwire collects data on the carrier country of the phone being used to complete the surveys, the team noted that all 10 of these participants’ phone carrier countries were Nigeria. All other participants enrolled in the study were from within the US time zones and had a phone carrier country listed as the United States. Notably, all 10 of these participants had presented a medical record to confirm their HIV diagnosis, while the wider study population tended to use a pill bottle. Overall, 2 of the 10 individuals only provided the first name of an alternative contact, and 3 provided alternative contacts whose names fit the naming convention (ie, common first name for both first and last names) that was noted by RAs to be suspicious in prescreening forms.

Supplemented by the patterns identified in prescreening forms, phone screening, and baseline assessment processes, the phone carrier country became the gold standard used by the study team to confirm authenticity or fraudulence among enrolled study participants. This standard could only be applied to persons who had already enrolled and reached the EMA portion of the trials.

## Addressing Enrolled Fraudulent Participants

Study activities for these individuals were immediately paused, and investigators notified the IRB. The 10 fraudulent participants were disenrolled from the trial. In October 2023, of the 10 individuals, 4 had been randomized and were engaged in the study intervention period, while the other 6 individuals were either actively engaged in EMA or had completed EMA and were awaiting randomization. The project manager emailed the individuals to inform them of their ineligibility and disenrollment and, at the recommendation of the IRB, provided any compensation to which they were entitled for completing the EMA surveys. Two individuals responded to this notice; one thanked the study team for the information and asked a question about compensation, and the other asked for an explanation for their dismissal, to which the study team responded that we were not able to share the ineligibility criteria and that, per their consent form, their participation could be terminated at any time.

## Development, Implementation, and Evaluation of Fraud Detection and Prevention Procedures

Based on the clues identified in the review of all enrolled participants believed to be fraudulent, we modified our prescreening, phone screening, and consent protocols and procedures (Table 1).

**Table 1:** Fraud detection and prevention strategies implemented.

Tool			Features
**Prescreening checklist**			
	Name	The name provided matched previous patterns (ie, first and last names were both common first names)	
	Email	The email provided matched previous patterns (ie, first name and last name followed by a string of numbers)	
	Age	The age provided matched previous patterns (ie, 20-35 years)	
	Area and ZIP code	The area and ZIP code provided did not correspond to the same state	
	Sex at birth	The sex at birth provided matched previous patterns (ie, male)	
	Level of physical activity	The level of physical activity provided matched previous patterns (ie, exceeding 150 minutes per day)	
	Interest	The level of interest provided matched previous patterns (ie, 5 out of 5 for both trials)	
	Availability	The amount of availability to be outreached matched previous patterns (ie, total availability every day)	
**Screening checklist**			
	Google Voice number	The potential participant used a phone number that was found to be a Google Voice number	
	Mismatched ZIP code	The potential participant reported a ZIP code that did not match the ZIP code they provided on the prescreening form	
	Urgent emails or call backs	The potential participant sent emails that were structured similarly to previously received emails with high urgency for response, or left voice messages with high urgency for response	
	Similar voice	The potential participant’s pattern of speech or accent was similar to previous individuals who were deemed potentially fraudulent	
	Quick answers	The potential participant responded quickly to questions that tended to require more thought or consideration (eg, average number of minutes exercised per week)	
	Follows script	The potential participant responded in predictable ways (eg, consistently saying “no questions” when asked)	
	Notes	Study staff wrote notes about other patterns of behavior that were uncovered during the screening process, not captured by the checklist items	
**Videoconference screening^a^**			
	Duplicates	If an individual being screened was identical or nearly identical in appearance to another participant previously enrolled	
	Misrepresenting personal or health history	Looking away from the camera after each screening question, turning off their camera, and muting their microphone frequently after screening questions, as if to request an answer from someone off camera	
**Baseline interview**			
	Government-issued photo identification	Study staff had the ability to ask for a photo ID if the staff member was concerned about authenticity	

^a^Potential participants were asked to complete the phone screening by videoconference if the study staff was suspicious of the individual’s authenticity. Videoconferencing at this screening stage helped study staff to evaluate whether individuals were potentially duplicates or misrepresenting their personal or health history.

One such modification was the implementation of a checklist for study staff to use when suspicions surrounding possible fraud arose. This checklist assisted staff during the prescreening and phone screening process in assessing a potential participant’s authenticity. All potential participants, regardless of concern about fraudulence based on their online prescreen responses, were contacted for phone screening if they met initial eligibility criteria. Research staff used the checklist (which was a list of characteristics associated with potential participant fraudulence) and took notes of any other patterns or concerning responses that surfaced at the phone screening stage. Potential participants who presented in a manner consistent with our “fraud detection” checklist were marked for principal investigator (PI) review. Upon review of this information, the PI would decide whether to deem the participant ineligible or invite them for a consent and baseline assessment.

For phone screening, we amended our protocol to conduct “phone” screens by videoconference rather than phone only in cases where there were markers of possible fraud at prescreening. When possible, the same staff member conducted all videoconference screening calls in order to support the identification of duplicate enrollment attempts. Video calls allowed research staff to identify duplicate or potentially fraudulent individuals who attempted to enroll by providing information about the individual’s appearance and behavior while answering screening questions (eg, frequently looking away from the camera as if to get the answer from someone else).

At the consent and baseline assessment contact, we amended the study protocol to require participants to provide a government-issued photo identification during the consent process if requested by study staff to verify identity and prevent participants from enrolling more than once. After further waves of suspected fraudulent activity occurred in February-April 2024, the study team amended the study protocol to require potential participants to provide this identification at the screening stage, if requested by study staff. Table 1 describes each strategy the study team implemented to identify and prevent fraudulent participants and activity.

Since the initial wave of fraudulent participants identified in October 2023, additional potentially fraudulent persons have attempted to enroll in the trials. Between November 2023 and November 2024, a total of 9 individuals who completed phone or videoconference screening for the PA trial and 28 individuals who completed phone or videoconference screening for the Pain trial were deemed fraudulent (though without Metricwire confirmation, which was unavailable as EMA was not performed during these screening stages) by study PIs after reviewing the checklist and notes compiled by study staff who screened the individuals. These 37 individuals were separate from the 10 who were disenrolled from the Pain trial, and none of these participants were consented and enrolled in the trials. To confirm the effectiveness of our fraud prevention procedures since we implemented them, we also examined phone carrier country information in Metricwire for all participants enrolled since we implemented our fraud prevention methods and found 0 with a non-US phone carrier or in a non-US time zone.

As part of an additional examination of our fraud prevention procedures, we compared the fraud indicator prescreening checklist responses submitted by the 10 confirmed and enrolled fraudulent participants with the other 101 authentic enrolled participants (those who met our gold standard confirmatory measure of US phone carrier in the Metricwire app) who had been enrolled at the time these 10 were identified (Table 2).

**Table 2:** Percentage of enrolled participants possessing suspected fraudulence indicators at the prescreening stage, overall, and by authenticity or fraudulent status.

Indicator	Total (n=111), n (%)	Authentic participants (n=101), n (%)	Fraudulent participants (n=10), n (%)
Name	15 (13.5)	8 (7.9)	7 (70)
Email address	14 (12.6)	11 (10.9)	3 (30)
Age (20-35 years)	33 (29.7)	25 (24.8)	8 (80)
Area and ZIP code mismatch	26 (23.4)	16 (15.8)	10 (100)
Male sex	102 (91.9)	94 (93.1)	8 (80)
High physical activity	7 (12.3)^a^	2 (4.3)^a^	5 (50)
High interest	28 (25.2)	21 (20.8)	7 (70)
High availability	18 (16.2)	11 (10.9)	7 (70)

^a^Authentic participants enrolled in the Increasing Physical Activity Among People Living With HIV Engaged in Unhealthy Drinking trial (PA trial; n=54) were not included in the high physical activity indicator percent calculation, as individuals displaying this characteristic at prescreening are not eligible for the PA trial and are thus not enrolled. Only participants enrolled in the Integrated Telehealth Intervention to Reduce Chronic Pain and Unhealthy Drinking Among People Living with HIV trial (n=57 total; n=47 authentic) were included in this calculation. Including authentic PA trial participants would artificially deflate this value.

The audit of these data suggests that all but one indicator (male sex) initially identified by the study team in October 2023 were found more frequently in those who were determined to be fraudulent. Notably, 100% (10/10) of fraudulent individuals who were disenrolled from the study had an area code and ZIP code that did not match the same state in their prescreening form, while only 15.8% (16/101) of authentic individuals possessed this indicator. These data suggest that the patterns found in prescreening forms can serve as an early indication of fraudulent activity.

## Discussion

Fraudulent participation in online studies is likely an underidentified problem in this new era of virtual recruiting. We describe our experiences in 2023 and 2024 when screening and enrolling people with HIV from across the United States into 2 linked randomized trials that do not include any in-person research visits. We discovered the attempted infiltration of these studies by what appeared to be an organized group of ineligible persons, an organized group of individuals from Nigeria, seeking study enrollment (and perhaps repeated participation) to receive financial remuneration. Unfortunately, we found this problem only after 10 persons were enrolled and 4 were randomized in our ARCHER Pain trial. Fortunately, we discovered the issue early after their enrollment, in time to disenroll them quickly, saving study resources and protecting data integrity. In this paper, we describe the development and implementation of methods to prevent enrolling additional ineligible, fraudulent participants. We believe, but cannot be certain, that the procedures we describe were effective.

We have provided a more comprehensive list of suspicious behaviors that may suggest the virtual research intrusion of persons using fake identities, although many of these signals of fraudulent activity have been noted before. Previous researchers have noted that unusual experiences during virtual interviews, such as individuals keeping their camera turned off, have been one of the first indicators of fakery [13,23]. Giving illogical answers or responses that are nearly identical to those offered by previous study participants has also alerted researchers to potential fraud [13,23]. Similar to our experience, Roehl and Harland [23] reported fraudulent participants using Google Voice phone numbers. Interestingly, fraudulent participants in their study also reported residing in New York City [23]; most of the fraudulent participants in our study reported living in New York as well. Other researchers have reported fraudulent participants who used email addresses that follow an unusual or consistent convention [2,23]. Fraudulent participation from outside the United States, which validated our suspicions, has also been previously observed [1].

Other investigators have noted that a sudden influx of screening form submissions or higher enrollment than projected has served as a signal that fraudulent activity may be underway [10,12-14]. After the initial wave of fraudulent activity in October of 2023 caused our study team to be more aware of potential fraudulence, we noticed that a change from the receipt of 2-3 prescreen form submissions per day to 10-12 per day became one of the first indications of attempted study infiltration. This rush of submissions was sometimes paired with forms submitted at unconventional times, such as between 3 AM and 5 AM, or numerous submissions within a short period of time. Other researchers have also noted suspicious submission times [10,11].

How can fraudulent enrollment in virtual studies be prevented? Certainly, the use of multiple methods concurrently is likely to be most effective, as each alone has limitations. Methods like CAPTCHA and reCAPTCHA used in previous research are excellent tools for removing nonhuman fraudulent activity [1,7-11]. However, they cannot be trusted to remove all robotic entries [11] and can be cost-prohibitive. Additionally, bot detection methods do not solve the human-driven fraudulent activity seen in this study. Tracking suspicious activity, such as short response times on screening forms or surveys, can be effective [6,8,11,12]. Our study did not originally have the capability to track the length of time spent completing the prescreen form, and due to the nature of fraudulence observed in our study and the short length of the prescreen form, this capability would have been less relevant. Using IP address checks to track when a single actor tries to submit multiple forms to enter a research study may be helpful [6-8]. A major drawback to relying on IP addresses to determine fraudulence is that a virtual private network can be used to evade IP tracking methods. Further, identical IP addresses may not indicate malicious intent but could rather be the result of being on the same computer network as another legitimate individual who tried to enroll in the study [9]. IP addresses may help researchers geolocate participants outside the study area, but this is also unreliable due to technologies such as remote access tools that link devices to a different IP address [2]. Additionally, tracking IP addresses, if not already built into an online recruitment platform, would require additional time and expensive web programming resources to implement.

Glazer et al [15] used an online identity verification tool to cross-reference the information submitted to their online screening portal. The tool used, TLOxp (TransUnion), pulls data from public sources to determine whether the name, birth date, and address obtained in the screening portal match what is publicly available. When considering whether to use such identity verification methods, researchers should consider the population being studied. Persons with HIV, a stigmatized diagnosis, may avoid screening altogether if they know their identity will be checked as part of the screening process.

Despite the benefits of automated fraud detection methods, manual methods should be used to fill in gaps, particularly when more flexible ways of detecting fraud are needed. Previous researchers found success through tracking characteristics and patterns of concern by creating a list of indicators that can categorize participation as fraudulent, suspicious, or authentic based on the number or type of indicators in each participant’s responses or data [2,7,11]. Manual methods of detection, such as these, can be time-consuming and burdensome compared to automated methods [6]. Yet manual methods allow for adaptability to changing patterns that fraudulent actors may pursue.

While at times inelegant, the use of the checklist we developed to monitor prescreen submissions and inauthentic attempts to join the research study allowed us to identify presumably fraudulent participants who would have been missed earlier in the study. Of course, it remains possible that fraudulent participants are still slipping past our detection methods and entering the trial. In addition, it is possible that our new procedures are overly sensitive and mistakenly rejecting authentic participants.

The addition of requesting a government-issued photo identification at the baseline assessment proved effective in verifying the authenticity of participants in our study and preventing duplicate enrollments, a strategy that previous researchers have also found effective [1,5]. When made aware of the requirement to show a photo identification, suspected fraudulent participants would often terminate the videoconference screening call or would fail to respond. Requiring a photo identification can help authenticate a participant’s identity and prevent duplicate enrollments.

The methods outlined here protected the privacy of research participants, particularly when dealing with vulnerable populations like people living with HIV. Screening procedures must balance the need to obtain more information to prevent fraudulent participation [13] and research accessibility, a primary benefit of virtual research. As threats of fraudulent research participation continue to emerge, researchers should collaborate with their IRBs and, when appropriate, funding agencies to develop and implement fraud identification and prevention methods that balance concerns about data integrity with regulatory compliance considerations such as protections of human participants’ rights and welfare and fiscal compliance. Upon discovering fraudulent participants in our trials, our research team promptly followed our institutional reporting policies and followed the IRB’s guidance on how to address fraudulent participant disenrollment and compensation. As others have recommended [24], researchers should consider engaging IRBs and funding agencies proactively in the study planning process to develop ethical approaches for handling cases of fraud should it occur, including procedures for compensation that appropriately balance participant rights while mitigating fiscal misuse.

### Conclusions

Fraudulent research participation is an ever-evolving aspect of virtual research, and vigilance is necessary to prevent it. Integrating multiple prevention methods during study start-up and continuing to review these methods in an iterative manner is critical [2]. Including automated methods can be an effective first step but should not be relied on alone. Because of the evolving nature of fraud attempts, we found that using a manual prescreen checklist in conjunction with phone and videoconference screening, as well as requesting that participants provide a government-issued photo identification to verify and confirm their identity, allowed us to identify and prevent fraudulent research participation in real time. Such manual methods used in combination with automated methods provide researchers with the information and tools necessary to identify and prevent fraud.

By reviewing and protocolizing prevention methods early and often, researchers can be better prepared to prevent and, if necessary, handle fraudulent participation when it happens. Each study’s experience of fraud is unique; it is important for researchers to share their experiences and forensic analysis so that virtual research can continue to adapt to new challenges.

## Abbreviations

ARCHER: Addressing Related Comorbidities for HIV by Employing Remote Technologies
EMA: ecological momentary assessments
IRB: institutional review board
PA trial: Increasing Physical Activity Among People Living With HIV Engaged in Unhealthy Drinking trial
Pain trial: Integrated Telehealth Intervention to Reduce Chronic Pain and Unhealthy Drinking Among People Living with HIV trial
PI: principal investigator
RA: research assistant
